# Bmal1 Regulates the Redox Rhythm of HSPB1, and Homooxidized HSPB1 Attenuates the Oxidative Stress Injury of Cardiomyocytes

**DOI:** 10.1155/2021/5542815

**Published:** 2021-06-18

**Authors:** Xiehong Liu, Wen Xiao, Yu Jiang, Lianhong Zou, Fang Chen, Weiwei Xiao, Xingwen Zhang, Yan Cao, Lei Xu, Yimin Zhu

**Affiliations:** ^1^Hunan Provincial Key Laboratory of Emergency and Critical Care Metabonomics, Institute of Emergency Medicine, The First Affiliated Hospital of Hunan Normal University (Hunan Provincial People's Hospital), Changsha, Hunan, China; ^2^Emergency Department, Hunan Provincial People's Hospital/The First Affiliated Hospital of Hunan Normal University, Changsha, Hunan, China; ^3^Public Health Clinical Center, Xiangtan Central Hospital, Xiangtan, Hunan, China

## Abstract

Oxidative stress is the main cause of acute myocardial infarction (AMI), which is related to the disorder of the regulation of Bmal1 on the redox state. HSPB1 form homologous-oxidized HSPB1 (homooxidized HSPB1) to resist oxidative damage via S-thiolated modification. However, it is still unclarified whether there is an interaction between the circadian clock and HSPB1 in myocardial injury. A total of 118 AMI patients admitted and treated in our hospital from Sep. 2019 to Sep. 2020 were selected to detect the plasma HSPB1 expression and the redox state. We divided the AMI patients into three subgroups: morning-onset AMI (5 : 00 am to 8 : 00 am; Am-subgroup, *n* = 38), noon-onset AMI (12 : 00 pm to 15 : 00; Pm-subgroup, *n* = 45), and night-onset AMI (20 : 00 pm to 23 : 00 pm; Eve-subgroup, *n* = 35) according to the circadian rhythm of onset. The Am-subgroup had remarkably higher cardiac troponin I (cTnI), creatine kinase MB (CK-MB), and B-type natriuretic peptide (BNP) but lower left ventricular ejection fraction (LVEF) than the Pm-subgroup and Eve-subgroup. Patients complicated with cardiogenic shock were significantly higher in the Am-subgroup than in the other two groups. The homooxidized HSPB1 in plasma markedly decreased in the Am-subgroup. The HSPB1C141S mutant accelerated H9c2 cell apoptosis, increased reactive oxygen species (ROS), and decreased reduced-glutathione (GSH) and the ratio of reduced-GSH and GSSG during oxidative stress. Importantly, we found that the redox state of HSPB1 was consistent with the oscillatory rhythm of Bmal1 expression in normal C57B/L mice. The circadian rhythm disorder contributed to decrease Bmal1 and homooxidized HSPB1 in cardiomyocytes of C57BL/6 mice. In addition, Bmal1 and homooxidized HSPB1 decreased in neonatal rat cardiomyocytes exposed to H_2_O_2_. Knockdown of Bmal1 led to significant attenuation in homooxidized HSPB1 expression, whereas overexpression of Bmal1 increased homooxidized HSPB1 expression in response to H_2_O_2_. Our findings indicated that the homooxidized HSPB1 reduced probably the AMI patients' risk of shock and target organ damage, which was associated with Bmal1 regulating the redox state of HSPB1.

## 1. Introduction

AMI is myocardial necrosis caused by acute, persistent ischemia, and hypoxia of coronary arteries, responsible for 16% of the world's total deaths [1, 2]. The imbalance of the myocardial redox state which induced the oxidative stress was the most important cause of cardiomyocyte dysfunction and injury [3]. Oxidative stress is the direct result affecting the outcome of thrombolytic therapy, angioplasty, and coronary artery bypass grafting in AMI [4]. Some studies have shown that the circadian clock in human heart tissue is particularly obvious, and the circadian disorder was a risk factor of cardiovascular disease [5–7]. Epidemiological data showed that the incidence of AMI showed a unique “morning peak” circadian rhythm [8, 9], which is related to the dysfunction of reactive oxygen species (ROS) scavenging caused by the decreased function of intracellular antioxidant defense system. Notably, the redox oscillation of endogenous antioxidants and circadian rhythm was consistent [10, 11]. Thus, further exploration of the redox and circadian may provide an in-depth insight into the mechanism in AMI.

Small heat shock proteins (sHSPs) are a protein superfamily that range in molecular size from 10 to 30 kDa and are present in all the major cellular compartments [12, 13]. HSPB1 is one of the most mature research sHSPs and generally is effective in the form of oligomer and phosphorylated dimers [14]. HSPB1 plays an important role in the resistance to oxidative stress by decreasing intracellular ROS and nitric oxide levels, maintaining a reduced glutathione (GSH) level, and stabilizing the mitochondrial membrane potential [15–17]. Our previous studies have shown that HSPB1 improved the reductive function of endogenous glutathione reductase (GR)/GSH/glutathione peroxidase (GPx) and thioredoxin (Trx)/peroxiredoxin (Prx) antioxidant system in rat cardiomyocytes, which further confirmed that HSPB1 was involved in protein oxidation resistance [18]. Kraemer et al. demonstrate that HSPB1 is upregulated and phosphorylated in platelets of patients with ST-elevation myocardial infarction [19]. Cardiomyocyte HSPB1 is required for wound healing after myocardial infarction (MI) and could be a target for myocardial repair in MI patients [20]. Recent reports demonstrated that secreted-type HSPB1 could be a potential index of atherosclerosis and biomarker of diabetic nephropathy [21, 22]. Significantly, HSPB1 formed homologous oxidized HSPB1 (homooxidized HSPB1) by its own unique cysteine and then played an antioxidant role in vitro [23]. In addition, the mutant cysteine of HSPB1 blocked the formation of homooxidized HSPB1 and changed the ability of HSPB1 to polymerize, then destroyed its activity of chaperone and phosphorylate activity [14, 24]. These studies have provided a foundation for exploring the potential roles of homooxidized HSPB1 in oxidative modification of HSPB1 in AMI.

The effects of oxidative stress and dysregulated circadian rhythms have been a subject of intense investigations in heart disease [25]. Several key enzymes and antioxidants, which include superoxide dismutase (SOD), catalase (CAT), Prx, GPx, GSH, GR, and glutathione S-transferases (GSTs), involved in protection from free radicals oscillate with circadian rhythmicity in the expression or activity level [26], suggesting that the clock system could regulate endogenous antioxidant systems. Most notably, the expression of the above-mentioned enzymes or antioxidants was the lowest in the early morning [10, 27], and the myocardial injury and dysfunction of early morning onset AMI was the most serious [28]. However, it is not clear whether the regulation of the circadian clock on endogenous antioxidant system is related to the onset time and severity of AMI. Bmal1 is a universal timing machinery of the circadian clock loop, which directs the sophisticated circadian expression of clock-controlled genes [29]. In recent years, some studies suggested that Bmal1 was a key transcription factor regulating oxidative response. Nakao et al. also found that ROS production increased in H9c2 cells with the BMAL1 gene knockout [30]. Bmal1^−/−^ mice show higher accumulation of ROS in several tissues compared with wild-type animals, and this impairment in ROS homeostasis correlates with the heart function [31, 32]. The study revealed the circadian control of the Nrf-2/GSH antioxidant pathway in combating oxidative fibrotic lung damage [33]. These data again suggest a connection between the circadian clock and redox homeostasis. Therefore, determining how this compartmentalized nature of cellular redox systems links to the clockwork will be critical to understand the cause of AMI in the early morning.

In the current study, we explored the mechanism of AMI from a new perspective of the circadian clock transcription factor Bmal1 regulating the endogenous antioxidant system via homooxidized HSPB1. We showed for the first time that the homooxidized HSPB1 could be used as a sensitizer of the intracellular antioxidant defense system, and the redox oscillation of HSPB1 has a circadian rhythm. Our research further revealed that a theoretical basis for AMI occurs in the early morning and most probably provided optimal intervention targets of AMI.

## 2. Methods

### 2.1. Human Blood Specimens

We performed this work after obtaining approval from the Institutional Ethics Committee of the Hunan Provincial People's Hospital (First Affiliated Hospital of Hunan Normal University) (Ethics Number: 2019S99). In our study, 118 patients aged more than 45 years from both sexes were recruited from the Hunan Provincial People's Hospital, Changsha, Hunan, China, after obtaining written informed consent from participants. Inclusion criteria: newly diagnosed AMI patients of both sexes admitted in the Intensive Care Unit with an elevated ST-segment of at least 2 mm in two or more consecutive leads of electrocardiography were included. Exclusion criteria: patients with a previous history of chronic angina or AMI, family history of heart and cerebrovascular diseases, on thrombolytic therapy, and unable to obtain informed consent were excluded. We evaluated circadian rhythms among the enrolled patients in terms of sleep, diet, and exercise by the Athens Insomnia Scale (AIS), Pittsburgh Sleep Quality Index (PSQ), and International Physical Activity Questionnaire (IPAQ). Patients were classified into three groups: group 1: morning-onset AMI (Am-subgroup); group 2: noon-onset AMI (Pm-subgroup); and group 3: night-onset AMI (Eve-subgroup) according to the pathogenic time of onset. The diagnostic criteria were derived from the American Heart Association (AHA). In all patients, medical history, physical examination, ECG, echocardiography, and chest X-ray as well as blood laboratory tests (blood count, electrolytes, glucose, hepatic and renal indicators, and heart indicator) were performed. The plasma of the patients was separated from blood specimens (centrifuged at 3000 rpm for 10 min) and saved in −80 °C till further analysis.

### 2.2. Estimation of HSPB1 by ELISA

The plasma levels of HSPB1 were measured with a Human HSP27/HSPB1 ELISA Kit (catalog# EK1244, Multi Sciences Biotech, Co., Ltd., Hangzhou, China) according to the manufacturer's protocol. Briefly, the precoated enzyme microplate was washed with wash buffer for 30 seconds. The diluted samples and horseradish peroxidase- (HRP-) labeled detection antibody (samples: antibody = 100 : 1) were added followed by incubation for 2 h at a culture temperature of 37°C and rotational speed of shaker of 300 rpm/min. The aspiration and wash step were repeated 6 times. The chromogenic substrate TMB was added, the plate was incubated for 30 min at room temperature avoiding direct light, and the reaction was stopped by adding stop solution. Thorough mixing was ensured upon addition of every reagent. The absorbance was measured using a microplate spectrophotometer set to 450 nm with wavelength correction set to 630 nm.

### 2.3. Animals

For mice, all the experiments were approved in advance by the Institutional Animal Care and Use Committee of Hunan Provincial People's Hospital (First Affiliated Hospital of Hunan Normal University). Six-to-eight-week-old male C57BL/6 mice (18~22 g) were purchased from Hunan Silaike Jingda Experimental Animal Co. Ltd., Changsha, Hunan, China (animal qualification certification: No.1107271911006355). The mice were housed in a cage under room temperature of 20°C to 26°C, relative humidity levels of 30% to 70%, and 12 h light-dark cycles with ad libitum access to tap water and a certified pellet diet. The use of animals in experiments was in accordance with the national experimental animal use regulations.

### 2.4. Isolation and Culture of Neonatal Rat Cardiomyocytes

24-to-72-hour-old Sprague Dawley rats were purchased from the Hunan Silaike Jingda Experimental Animal Co. Ltd., Changsha, Hunan, China (animal qualification certification: 43072720110107434). Neonatal rat cardiomyocytes were isolated as previously described [34]. Briefly, hearts were isolated and transferred to Hank's Balanced Salt Solution (HPSS). The ventricles were then minced into small pieces, followed by digestion by trypsin (4 mg/mL) and collagenase P (1.0 mg/mL) at 37°C. Next, cardiomyocytes were recovered by centrifugation before they were resuspended in a growth medium supplemented with medium 199 (×1), 10% FBS, and 100 U/mL of penicillin and streptomycin. The cardiomyocytes were then incubated at 37°C and 5% CO_2_ in a humidified incubator. After 90 min, the supernatant was collected, and cardiomyocytes were then replated onto petri dishes at a concentration of 5 × 10^5^ cells/dish. After 48 h, the neonatal rat cardiomyocytes were washed and cultured in serum-free medium 199 at 37°C and 5% CO_2_ for the duration of the whole experiment.

### 2.5. GSH and Glutathione Disulfide Measurement

GSH and glutathione disulfide (GSSG) levels were measured using a kit (Cayman Chemical), which used a spectrophotometric GR recycling assay. Briefly, homogenize the tissue sample in 0.1% Triton-X in 0.1 M assay buffer (pH 7.5) ice cold. Centrifuge at 1000 × g for 5 min in 4°C to remove the debris. Collect the supernatant and save 10 *μ*L to perform protein determination. Add 2% sulfosalicylic acid (freshly prepared) and centrifuge at 1000 × g for 10 min in 4°C. Collect the supernatant. Then, add 2% sulfosalicylic acid (freshly prepared) and centrifuge at 1000 × g for 10 min in 4°C. Collect the supernatant. The acidified supernatants can be divided in two and used directly for total-GSH or derivatized for the GSSG assay according to the manufacturer's instructions. Importantly for the GSSG measurement, the samples must be mixed quickly with vinylpyridine (4 *μ*L of 1 : 10 diluted vinylpyridine in 0.1 assay buffer for 100 *μ*L of supernatant). After vortexing for 15 s, samples are incubated for 2 h at RT in a fume hood. To inactivate nonreacted vinylpyridine, add 6 *μ*L of triethanolamine (1 : 6 diluted in assay buffer) and incubate 10 min. All measurements were normalized to the protein content, as determined by a bicinchoninic acid (BCA) protein assay kit. The absorbance was recorded at 405 nm using a plate reader at 5 min intervals for 30 min.

### 2.6. Redox Western

For HSPB1 redox analysis, the cells were washed with ice-cold PBS after treatment immediately. Cells were precipitated with 10% chilled trichloroacetic acid (TCA) for 30 min at 4°C. Then, the samples were centrifuged (12,000 g for 10 min) and washed with 100% ice-cold acetone. The protein pellets were carboxymethylated in guanidine-Tris solution (6 M guanidine-HCl, 50 mM Tris, pH 8.3, 3 mM EDTA, and 0.5% (*v*/*v*) Triton X-100) containing 50 mM iodoacetic acid (IAA) and incubated for 30 min at 37°C. Excess IAA was removed by Sephadex chromatography (MicroSpin G-25 columns, Amersham Biosciences) after which samples were diluted in 53 nonreducing sample buffers (0.1 M Tris-HCl, pH 6.8, 50% (*v*/*v*) glycerol, and 0.05% (*w*/*v*) bromophenol blue) and separated on a nonreducing SDS-polyacrylamide gel electrophoresis (PAGE) on 15% gel. Gels were electroblotted to a polyvinylidene difluoride membrane and probed for HSPB1 using an anti-HSPB1 primary antibody (No: ADI-SPA-801-F; Enzo Life Sciences, Farmingdale, NY) and Alexa Fluor 680 nm anti-goat IgG secondary antibody (Molecular Probes, Eugene, OR). Immunoblot signals were visualized with a ChemiDoc™ Imaging System (BLM Biotechnology Co., Ltd.). Bands were quantified using the ImageJ software (NIH, Bethesda, MD). As described by Schafer and Buettner [35], redox potentials were determined using band intensities and the Nernst equation:

(1) $$\begin{array}{c} \text{Ehc}=-240-\left(\frac{59.1}{2}\right)\log\;\left(\frac{\left[\text{HSPB}1‐\text{SH}\right]2}{\left[\text{HSPB}1‐\text{S}‐\text{S}‐\right]}\right)\text{mV}\;\left(25^{{}^{\circ}}\text{C},\text{pH }7.0\right). \end{array}$$

### 2.7. ROS Measurement

ROS production was measured with the cell-permeable probe CM-H2DCFDA. The cells were plated 24 hr before the assay in six-well plates. The CM-H2DCFDA dye was loaded by incubation at a concentration of 10 *μ*M for 30 min at 37°C. After incubation, cells were washed twice with PBS and treated with H_2_O_2_. Fluorescence was quantified at 0, 15, 15, 30, 60, and 120 min of treatment with H_2_O_2_ using a flow cytometer (FACS Gallios; Beckman Coulter, Brea, CA) with excitation at 485 nm and emission at 530 nm. The threshold value was set at 5 min in the vector group, and subsequently, the change in threshold percentage of the fluorescence intensity of each group was analyzed.

### 2.8. Quantitative Reverse Transcription Polymerase Chain Reaction

Gene expression was determined by quantitative reverse transcription polymerase chain reaction (qRT-PCR) analysis. The total RNA was isolated from H9c2 cells using TRIzol (15596-026; Invitrogen), and then, the 1 *μ*g sample of RNA was reverse-transcribed using a PrimeScript RT Reagent Kit (RR047A; TaKaRa, Shiga, Japan) according to the manufacturer's instructions. The relative gene levels were determined by RT-qPCR using the StepOnePlus™ Real-Time PCR System (Applied Biosystems, USA). All RT-qPCR mixtures were prepared using an SYBR Premix Ex Taq kit (RR820A; TaKaRa) with specific primers (Table 1). The mRNA levels of all target genes were normalized to the expression of the housekeeping gene actin. Relative quantities were determined using the comparative *^ΔΔ^*Ct method.

### 2.9. Data Analysis

Baseline characteristics as continuous and categorical variables were presented as median (interquartile range) and *n* (%), respectively, and examined by *χ*^2^ test or Fisher's exact test where appropriate. A two-sided significance level of *p* = 0.05 was used to evaluate statistical significance. Each experiment was repeated three times. For Western blots, one representative image is shown. The results are presented as the means ± standard deviations. Statistical significance was analyzed using one-way analysis of variance and Tukey's multiple comparison tests, followed by data analysis with GraphPad Prism (La Jolla, CA). The comparison between groups was carried out by one-way and two-way ANOVA with Dunnett and Bonferroni posttests, respectively.

## 3. Results

### 3.1. Clinical Profile of Patients across the Groups

**Figure 1:**
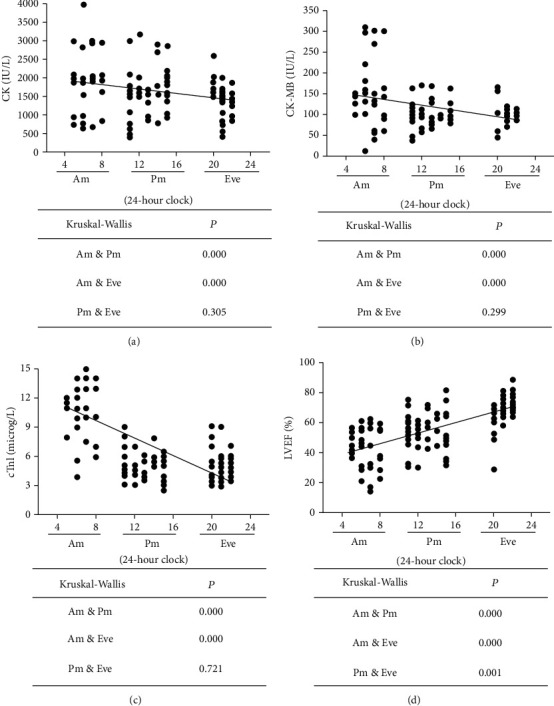
Pearson correlation and Kruskal-Wallis analysis of onset time effect on AMI.

In order to elucidate possible links between pathogenic time and severity, one hundred eighteen AMI participants completed the study. The participants were divided into three subgroups: 38 participants in the Am-subgroup, 45 participants in the Pm-subgroup, and 35 participants in the Eve-subgroup. None of the following parameters differed among the three groups: age, sex, BMI, smoking, drinking, diabetes mellitus, hypertension, hyperlipidemia, Scr, LDL-c, TG, TC, FPG, CRP, ALT, and AST levels (Table 2). Pearson correlation analysis showed that pathogenic time from morning to night was positively correlated with LVEF value (*r* = 0.6508, *p* < 0.001) and negatively correlated with CK, CK-MB and cTnI levels (*r* = −0.2401, -0.3756, -0.6618, *p* < 0.01). In addition, Kruskal-Wallis analysis indicated that patients in the Am-subgroup had markedly higher levels of CK, CK-MB, and cTnI compared to those in the Pm-subgroup and Eve-subgroup, and those in the Am-subgroup were more likely to display significantly lower EF values than those in the Pm-subgroup and Eve-subgroup (Figures 1(a)–1(d)). The above results suggested that cardiomyocyte injury and cardiac insufficiency were more serious in AMI patients with onset in the morning.

### 3.2. Homooxidized HSPB1 Was Downregulated in the Plasma during AMI Patient Onset in the Morning

**Figure 2:**
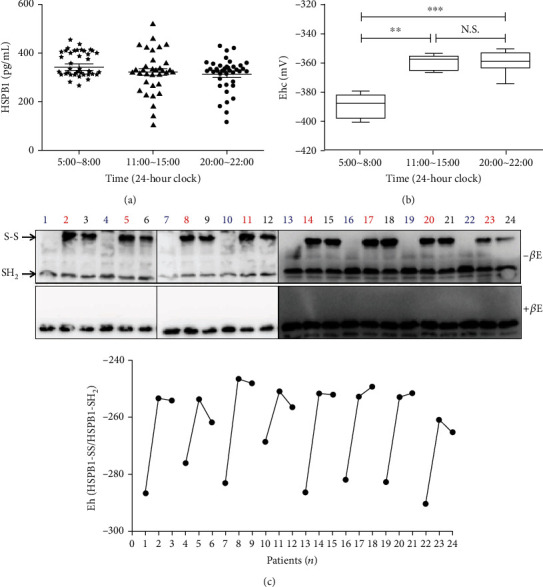
The expression of homooxidized HSPB1 was decreased in the plasma of AMI patients. (a) ELISA assay quantified the plasma of HSPB1, and the absorbance was measured using a microplate spectrophotometer set to 450 nm with wavelength correction set to 630 nm. *n* = 38 for the Am subgroup, *n* = 45 for the Pm subgroup, and *n* = 35 for the Eve subgroup. Error bars demonstrated means ± SD of 3 replications. ^∗∗^*p* < 0.01, ^∗∗∗^*p* < 0.0001. (b) The patient's plasma was diluted 5 times with sterilized water containing 100 mM NEM alkylating agent, and then, NEM-alkylated redox Western blot analysis was performed to detect the redox state of HSPB1. For reducing SDS-PAGE, 5% dithiothreitol was added to the samples. (c) Redox potentials were determined using band intensities and the Nernst equation. *n* = 8 for each group; blue, red, and black numbers on behalf of Am subgroup, Pm subgroup, and Eve subgroup, respectively. ^∗∗^*p* < 0.01 and ^∗∗∗^*p* < 0.0001.

Plasma phosphorylated HSPB1 has been reported to be involved in the pathogenesis of several diseases such as atherosclerosis [21] and chronic kidney disease [22]. Of note, the cysteine in the N-terminal domain is important for the equilibrium between HSPB1 oligomers and dimers [36]. Based on our above research, the time of AMI was related to the injury of cardiomyocytes; we firstly detect whether the expression of plasma HSPB1 changed in patients with different onset times. However, the ELISA results demonstrated that the level of HSPB1 was no different among the three groups (Figure 2(a)). Subsequently, we randomly selected 8 patients from each of the three subgroups and diluted the plasma samples with sterilized water containing alkylating agents, then detected the redox state of plasma HSPB1 using nonreducing SDS-PAGE. As shown in Figures 2(c) and 2(d), the decreased oxidized form of HSPB1 was observed in AMI patients with onset in the morning, whereas the total amount of HSPB1 was not different under reducing conditions.

### 3.3. The Homooxidized HSPB1 Resisted Oxidative Stress Damage Induced by H_2_O_2_ in H9c2 Cells

**Figure 3:**
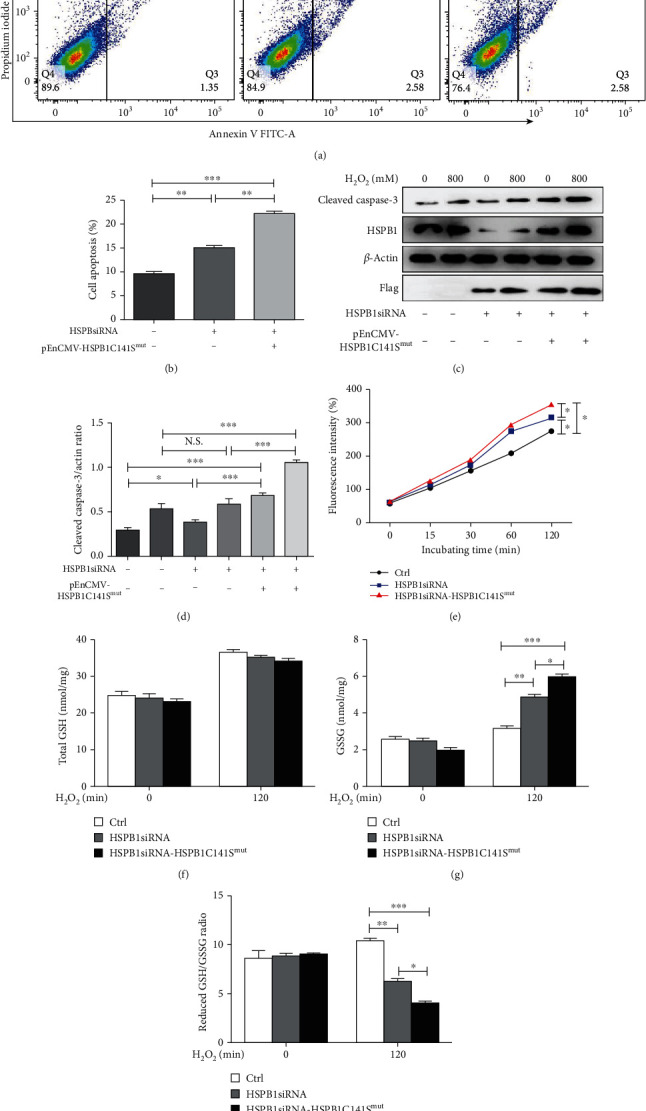
Homooxidized HSPB1 was necessary for HSPB1 to resist oxidative stress damage. (a, b) The pEnCMV-HSPB1C141S^mut^ or vector was transfected into the H9c2 cells with HSPB1 stable knockout for 48 h; flow cytometric analysis of cell apoptosis was conducted. (a) Representative plots of flow cytometric analysis are presented. (b) Data are presented as the means ± standard deviation (*n* = 6). (c–f ) The pEnCMV-HSPB1C141S^mut^ or vector was transfected into the H9c2 cells with HSPB1 stable knockout for 48 h; (c, d) cells were then treated with 800 mM H_2_O_2_ for 24 h. Western blot analysis was used to determine the levels of cleaved caspase-3; (e) H9c2 cells transfected with pCMV‐myc‐HSPB1 or vector for 48 hr and then incubated with CM‐H2DCFDA dye at a concentration of 10 *μ*M for 30 min at 37°C. Cells were then treated with 0.4 mM H_2_O_2_ for the indicated time periods (15, 30, 60, and 120 min). Fluorescence was quantified using flow cytometry with excitation at 485 nm and emission at 530 nm (^∗^*p* > 0.05). (f–h) Intracellular total GSH (f) and GSSG (g) levels were determined by glutathione reductase recycling assays. The reduced GSH/GSSG ratio (h) was calculated using reduced and oxidized GSH concentrations. Values represent the means ± standard deviations (*n* ≥ 6; ^∗^*p* < 0.001; ^#^*p* < 0.001).

The above results showed that homooxidized HSPB1 in plasma was related to the severity of AMI; we investigated the role of homooxidized HSPB1 in H9c2 cells exposed to oxidative stress. We first constructed the HSPB1 stable knockout H9c2 cell line (Figure S1A-B) and the HSPB1C141S mutant (pEnCMV-HSPB1C141S^mut^) plasmid (Figure S1C-E). Then, we observed the effect of the HSPB1C141S mutant on H_2_O_2_-induced oxidative stress injury in the HSPB1 stable knockout H9c2 cell line. Flow cytometry-based results revealed that the HSPB1C141S mutant significantly increased the rate of cell apoptosis (Figures 3(a) and 3(b)). Additionally, our results showed that the HSPB1C141S mutant also significantly improved cleavage of caspase-3 in response to H_2_O_2_ in H9c2 cells (Figures 3(c) and 3(d)). To further elucidate the effect of the HSPB1C141S mutant, we investigated the level of ROS, and the changes in GSH. The HSPB1C141S mutant induced the accumulation of ROS in H9c2 cells of H_2_O_2_-induced oxidative stress injury (Figure 3(e)). As shown in Figures 3(f)–3(h), the HSPB1C141S mutant did not affect the levels of total GSH but increased the level of GSSG and reduced the ratio of reduced GSH to GSSG. These results indicated that the formation of homooxidized HSPB1 was necessary for HSPB1 to resist oxidative stress.

### 3.4. The Redox Oscillation of HSPB1 and the Expression of Bmal1 Were Consistent

**Figure 4:**
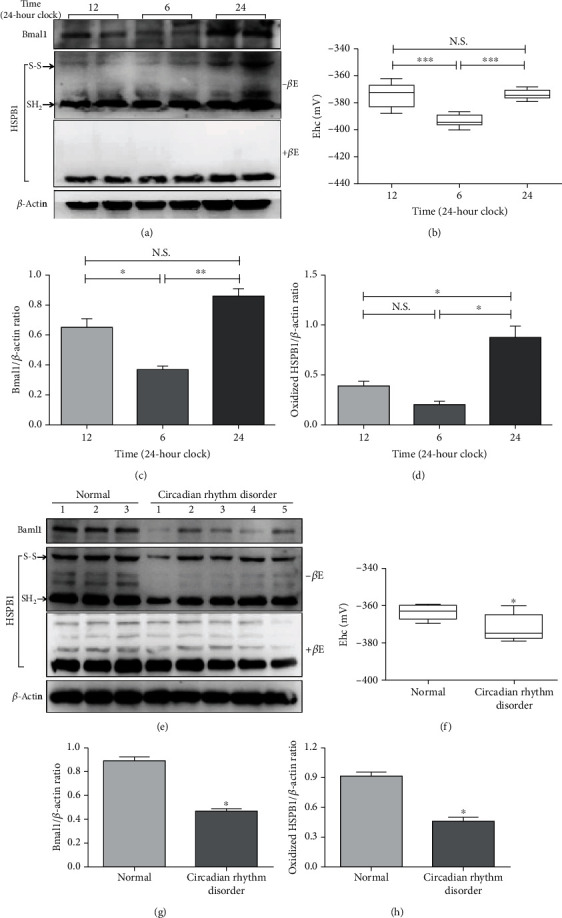
Circadian rhythm disorder decreases the level of homooxidized HSPB1 and Bmal1 in C57BL/6 mice. (a–d) A piece of ventricular muscle coming from the apex of a mouse heart was extracted at 12, 6, and 24 o'clock (24-hour clock), and two mice were collected from each group each day for a total of 7 days (*n* = 14 for each group). (e–h) The C57BL/6 mice in the experimental group were treated by day-night reversal for 14 days, namely, 6:00 to 18:00 every day as nonlight time (24-hour clock). (a, e) Bmal1 and *β*-actin were measured by Western blot analysis, and the homooxidized HSPB1 was analyzed by NEM-alkylated redox Western blot. (b, f) HSPB1 redox potentials were determined using band intensities and the Nernst equation. Densitometry was used to determine the fold expression of Bmal1 (c, g) and homooxidized HSPB1 (d, h) compared with *β*-actin. (a–d) *n* = 6 for each group; (e–h) *n* = 5 for each group; ^∗^*p* < 0.05, ^∗∗^*p* < 0.01, and ^∗∗∗^*p* < 0.001.

The work mentioned above suggested that the expression of homooxidized HSPB1 was different in AMI with onset at different times, suggesting that there may be a time rhythm in the redox state of HSPB1. Bmal1 is a principal driver of a molecular clock in mammals, and Bmal1 deletion abolishes 24-hour activity patterning [37]. Therefore, in order to explore the possible relationship between homooxidized HSPB1 and Bmal1, we first observed the expression of two of them in normal C57BL/6 mice. As demonstrated in Figures 4(a) and 4(b), the total amount of HSPB1 was not different under reducing conditions, whereas redox Western blots indicated that the relative expression level of homooxidized HSPB1 was highest at 24 o'clock and significantly lowest at 6 o'clock. Importantly, the oscillation expression of homooxidized HSPB1 in one day was similar to that of Bmal1 (Figures 4(a) and 4(c)).

The above results suggested that Bmal1 may be related to the redox state of HSPB1. Therefore, we further observed if the redox oscillation of HSPB1 in day-night reversal induced the circadian clock disorder of C57BL/6 mice. Our results showed that the circadian rhythm disorder obviously not only reduced Bmal1 protein expression but also significantly decreased the level of homooxidized HSPB1 (Figures 4(e)–4(h)). Taken together, our data suggested that the redox oscillation of HSPB1 and the expression of Bmal1 were consistent.

### 3.5. Bmal1 Was Involved in Regulating the Redox State of HSPB1 in Oxidative Stress Cardiomyocytes

**Figure 5:**
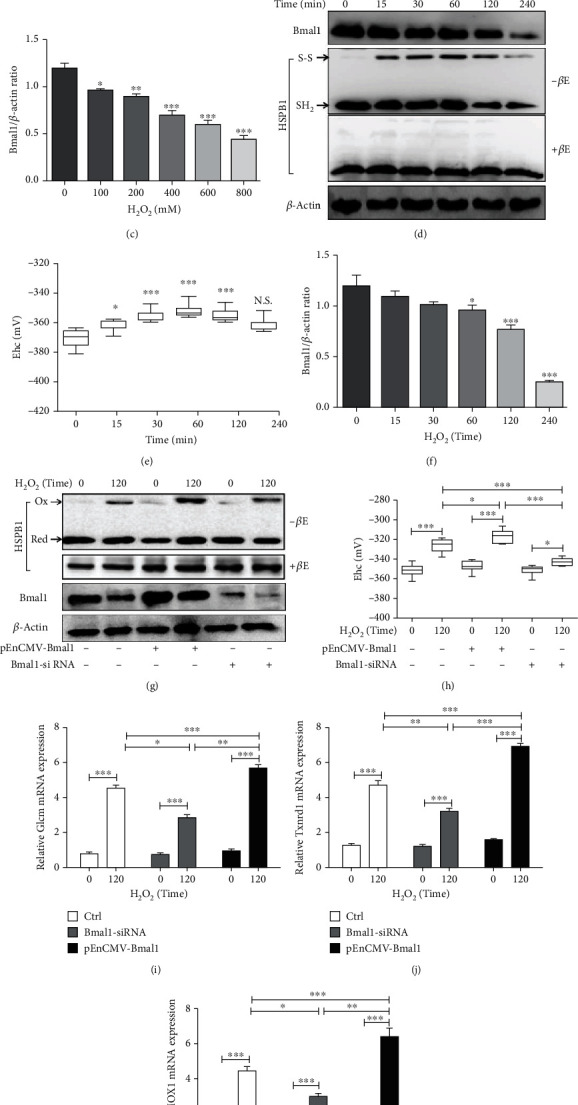
Bmal1 increased the level of homooxidized HSPB1. (a–f) NRCM isolation and culture methods were mentioned in Methods. (a–c) NRCMs were treated with H_2_O_2_ at 0, 100, 200, 400, 600, and 800 mM for 120 min, and (d–f) NRCMs were treated with 800 mM of H_2_O_2_ for 0, 15, 30, 60, 120, and 240 min; (a, d) Bmal1 and *β*-actin were measured by Western blot analysis, and the homooxidized HSPB1 was analyzed by NEM-alkylated redox Western blot. (b, e) HSPB1 redox potentials were determined using band intensities and the Nernst equation. (c, f) Densitometry was used to determine the fold expression of Bmal1 compared with *β*-actin. (g–k) H9c2 cells were transfected with 2 *μ*g pCMV-myc-Bmal1 or 15 nM Bmal1-siRNA for 48 h and then treated with different concentrations of H_2_O_2_ (0 or 800 mM) for 120 min. (g) NEM lysis buffer was used for extraction of total protein. (h) HSPB1 redox potentials were determined using band intensities and the Nernst equation. Relative mRNA expression of Glcm (i), Txnrd1 (j), and HMOX1 (k) in H9c2 cells compared with the control. Graphs are representatives of three independent experiments. *n* ≥ 6; ^∗^*p* < 0.05, ^∗∗^*p* < 0.01, and ^∗∗∗^*p* < 0.001.

The molecular clock is essential for cell survival after critical damage [38], and oxidative stress formation has been closely associated with the clock disturbance [39]. Thus, we observed the level of Bmal1 in the AMI rat model. Compared with the sham group, the expression of Per mRNA and Cry2 mRNA in the ischemic area of the heart of the AMI group and the myocardial I/R group increased significantly, while the expression of BMAL1 mRNA decreased (Figure S2A). As shown in Figure S1B, the Bmal1 protein expressions were obviously decreased in the AMI group and the I/R group. Combining with the above-mentioned research, we also observed the Bmal1 expression and redox state of HSPB1. Both the mRNA and the protein level of HSPB1 were increased in the AMI group and myocardial I/R group (Figure S2A and 1B). But, the nonreducing immunoblotting indicated that the expression of homooxidized HSPB1 was downregulated in the AMI group and myocardial I/R group (Figure S2C). Similarly, H_2_O_2_ obviously decreased the Bmal1 expression in a dose-dependent and time-dependent manner in NRCMs. Of note, when 600 mm H_2_O_2_ treated NRCMs for 2 h, the formation of homooxidized HSPB1 decreased (Figures 5(a)–5(f)). These results further indicated that Bmal1 and homooxidized HSPB1 are related to myocardial oxidative stress injury. Therefore, we sought to determine whether Bmal1 was responsible for the regulation of the redox of HSPB1 in cardiomyocytes exposed to oxidative stress. As shown, a higher expression of Bmal1 remarkably upregulated the expression of homooxidized HSPB1. Besides, the knockdown of Bmal1 decreased the expression of homooxidized HSPB1 (Figures 5(g) and 5(h)). In addition, overexpression of Bmal1 caused a significant increase in mRNA levels of Nrf-2-target genes, including Glcm, Txnrd1, and HMOX1, compared with those in control during oxidative stress. Conversely, siRNA-mediated knockdown of Bmal1 significantly reduced the expression of these target genes (Figures 5(i) and 5(j)). These data supported the regulation of Bmal1 on homooxidized HSPB1 expression in oxidative stress injury.

## 4. Discussion

Etiopathogenesis of myocardial infarction (MI) showed that the onset significantly varies throughout the day, which relates to impairment in redox regulation and circadian rhythms [40, 41]. However, there had been very little research focused on the interaction between the redox and circadian. HSPB1 forms S-thiolated modification without phosphorylation during oxidative stress and then disaggregates multimeric HSPB1 [42, 43]. But the research on homooxidized HSPB1 was not clear yet. In the present study, we uncovered that the circadian protein Bmal1 and homooxidized HSPB1 had played important roles in AMI. We had shown for the first time that downregulated homooxidized HSPB1 was observed in the plasma specimens of AMI patients with onset in the morning time. The homooxidized HSPB1 played an important role in resisting oxidative stress injury in H9c2 cardiomyocytes. Also, the oscillation expressions of homooxidized HSPB1 and Bmal1 were consistent in the mice as well as NRCMs with H_2_O_2_ exposure. Besides, Bmal1 regulated the redox state of HSPB1 with H_2_O_2_ exposure. Our results suggested that Bmal1 might hold out the oxidative stress though upregulation of the homooxidized HSPB1.

It is generally true that small heat shock proteins (SHPs) contain a very few or no cysteine residues. This is thought to give them the flexibility of conformational folding and refolding, which is necessary in their chaperone function [44]. HSPB1 only has a cysteine residue in the *α*-crystallin domain at position 137 (human) or 141 (murine). During cardiac ischemia injury, HSPB1 is S-thiolated and form homooxidized HSPB1 [45, 46]. Some studies have proven that homooxidized HSPB1 regulated the HSPB1 multimeric aggregate size independently of phosphorylation, and phosphorylation did not affect the stability of homooxidized HSPB1 [14, 24, 47]. The above studies indicate that homooxidized HSPB1 may be involved in the antioxidation, antiapoptosis, and anti-inflammatory effects of HSPB1. Similar to the previous data, we found that the total expression of the plasma HSPB1 had no significant difference among the AMI patients with onset at different times. While in the Am-subgroup patients with the most severe myocardial injury, the plasma homooxidized HSPB1 was reduced significantly. In addition, our results also showed that homooxidized HSPB1 resisted oxidative stress damage in cardiomyocytes. The above results indicated that the expression of plasma homooxidized HSPB1 reflected a certain degree of oxidative stress injury in AMI patients. But more studies are needed to evaluate the clinical value of homooxidized HSPB1 in more AMI patients.

Cell-autonomous circadian rhythms are particularly related to the cardiovascular system. The circadian rhythm mechanism coordinates the rhythms of heart rate, blood pressure, cardiac contractility, metabolism, and gene and protein abundance in a 24-hour circadian cycle [48]. On the contrary, disrupting the circadian rhythm (such as shift work and sleep disorders) increases the risk of cardiovascular disease, aggravates heart remodeling, and worsens the outcome. ROS is an important factor that causes complications, especially pathophysiological damage after myocardial infarction [49, 50]. Kohsaka et al. reported that loss of function of Bmal1 induced expression of genes related to oxidative stress, cardiac remodeling, and inflammation [51]. In the present study, our results further support the notion that Bmal1 ensures cardioprotection via resisting the oxidative stress.

More recently, time-of-day-dependent regulation of Nrf-2 has been reported, which takes an important role for antioxidants [36, 52, 53]. Chhunchha et al. reported that the molecular Bmal1 controls the inflammatory response through regulating Nrf-2 in innate immune cells [54]. Mahajan et al. found that Bmal1 and Nrf-2 cooperatively control oxidative response and redox homeostasis by regulating the rhythmic expression of Prx [31]. Base on the above-mentioned research, the links between circadian physiology and prooxidizing changes in the redox state, as reflected by a decline in redox potential, seem to be coherent and well established. However, we know very little about how the circadian rhythm mechanism participates in pathological oxidative stress in cardiac myocyte injury. In our studies, we first found that the redox oscillation of HSPB1 was in line with Bmal1 and further found that Bmal1 upheld the level of homooxidized HSPB1 and increased the mRNA levels of Nrf-2-target genes. Importantly, the result of Figure S3A and B showed that homooxidized HSPB1 promoted the transcription of Nrf-2 to the nucleus. Taken together, our findings suggesting a causal relationship between Bmal1 and the homooxidized HSPB1 to resist the oxidative stress injury in cardiomyocytes, which might be related to enhance the function of Nrf-2. Consequently, we will proceed to investigate the mechanism in subsequent experiments.

This study further supports the notion that the circadian clock protein Bmal1/homooxidized HSPB1/Nrf-2 pathway might be a new signaling pathway to alleviate myocardial injury. It must be noted that our clinical findings could be regarded as preliminary due to the rather small sample, and replication is needed in a large cohort of AMI patients. Nevertheless, the regulation of the circadian rhythm of HSPB1 may be targets for therapeutic intervention in patients with AMI.

## 5. Conclusions

**Figure 6:**
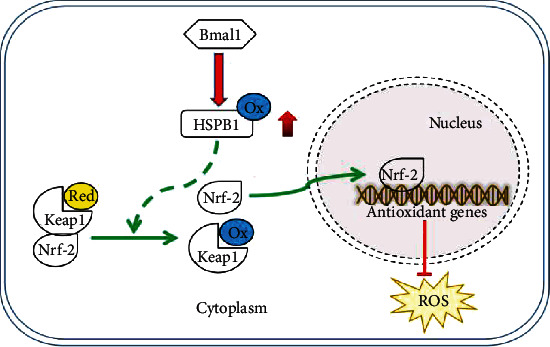
Schematic illustrating the working principle of the time rhythm of HSPB1 redox on oxidative stress injury. We propose that Bmal1 maintained the oxidized state of HSPB1, thereby promoting the antioxidant function of HSPB1. Additionally, homooxidized HSPB1 might increase the Nrf-2 nuclear localization, thereby driving the transcription of genes associated with cytoprotection. Ox-HSPB1: homooxidized HSPB1.

In summary, our findings identified a previously unrecognized role for homooxidized HSPB1 in the regulation of endogenous antioxidant pathways and established a relationship between homooxidized HSPB1 and the circadian system (Figure 6).

## Figures and Tables

**Table 1:** Primer sequence of RT-PCR.

Gene	Primer sequence (5′-3′)	
*TXNRD1*	Sense	GGATTCCTGGCTGGTATCGG
	Antisense	TTGTGGACTTAGCGGTCACC
*GCLM*	Sense	TTAGTTCAGAGCAAGAAGATTGT
	Antisense	TTACTATTGGGTTTTACCTGTGCC
*HMOX1*	Sense	CTCATCCTGAGCTGCTGGTG
	Antisense	GATGCTCGGGAAGGTGAAAA

**Table 2:** Clinical characteristics of the three groups.

Characteristics	Am-subgroup, *n* = 38	Pm-subgroup, *n* = 45	Eve-subgroup, *n* = 35	*p* value
Age (mean ± SD years)	60.12 ± 10.01	59.69 ± 11.21^∗^	62 ± 10.23^#^	0.963^∗^; 0.831^#^
BMI (kg/m^2^)	24.18 ± 8.81	24.10 ± 2.89^∗^	24.79 ± 2.24^#^	0.992^∗^; 0.933^#^
Female, *n* (%)	10 (26.3)	12 (26.7)	9 (25.7)	0.898
Smoker, *n* (%)				0.952
Never	10 (26.3)	12 (26.7)	9 (25.7)	
Past	13 (34.2)	12 (26.7)	10 (28.5)	
Current	15 (39.5)	21 (46.7)	16 (45.7)	
Drinker, *n* (%)	20 (52.6)	18 (40.0)	17 (48.6)	0.497
Hypertension, *n* (%)	9 (23.7)	10 (22.2)	8 (22.9)	0.987
Hyperlipidemia, *n* (%)	5 (13.2)	7 (15.6)	3 (11.4)	0.514
Diabetes mellitus, *n* (%)	3 (7.9)	5 (11.1)	2 (5.7)	0.682
FPG (mmol/L)	6.76 ± 2.21	7.12 ± 1.10^∗^	6.58 ± 2.14^#^	0.868^∗^; 0.949^#^
CRP (mg/L)	18.11 ± 12.37	17.45 ± 13.42^∗^	16.98 ± 15.14^#^	0.969^∗^; 0.553^#^
TC (mmol/L)	4.50 ± 0.89	4.52 ± 0.75^∗^	4.28 ± 1.02^#^	0.985^∗^; 0.099^#^
TG (mmol/L)	1.36 ± 0.75	1.27 ± 1.08^∗^	1.47 ± 0.62^#^	0.940^∗^; 0.280^#^
HDL-C (mmol/L)	1.15 ± 0.10	1.35 ± 0.21^∗^	1.28 ± 0.45^#^	0.380^∗^; 0.586^#^
LDL-C (mmol/L)	2.71 ± 0.87	2.85 ± 0.22^∗^	2.64 ± 0.36^#^	0.845^∗^; 0.931^#^
ALT (U/L)	48.29 ± 18.24	50.31 ± 17.95^∗^	49.26 ± 15.98^#^	0.007^∗^; 0.535^#^
AST (U/L)	185.64 ± 123.11	179.12 ± 98.77^∗^	162.45 ± 103.45^#^	0.689^∗^; 0.756^#^

Data are presented as mean ± SD or number (percentage). Am-subgroup: morning-onset AMI; Pm-subgroup: noon-onset AMI; Eve-subgroup: night-onset AMI. Smoker: Past—the patient's smoking-quitting exceeds three months but had smoked at least five cigarettes a day in the past two years or nonsmokers within three months; Current—the patients had smoked at least five cigarettes a day for two years. Drinker: drinks more than 4 drinks a day. FPG: fasting plasma glucose; SCR: serum creatinine; CRP: C-reactive protein; TC: total cholesterol; TG: triglyceride; HDL-C: high-density lipoprotein cholesterol; LDL-C: low-density lipoprotein cholesterol; ALT: alanine aminotransferase; AST: aspartate transaminase. ^∗^Pm-subgroup vs. Am-subgroup; ^#^Eve-subgroup vs. Am-subgroup.

## Data Availability

The laboratory (including animals and cells) data used to support the findings of this study are included within the article. The laboratory data used to support the findings of this study are included within the supplementary information file(s). The clinical data used to support the findings of this study are restricted by the Institutional Animal Care and Use Committee of Hunan Provincial People's Hospital in order to protect the patients. Data are available from the contact details for researchers who meet the criteria for access to confidential data.

## Supplementary Materials

Supplementary Materials Figure S1: constructed from the HSPB1 stable knockout H9c2 cell line and the HSPB1C141S mutant plasmid. Figure S2: the expression of Bmal1 and homooxidized HSPB1 decreased in AMI rat model. Figure S3: homooxidized HSPB1 promoted nuclear transcription of Nrf-2 in H9c2 cells exposed in H_2_O_2_.
