# Supplementary material for: Bmal1 Regulates the Redox Rhythm of HSPB1, and Homooxidized HSPB1 Attenuates the Oxidative Stress Injury of Cardiomyocytes
Source: Oxid Med Cell Longev. 2021 Jun 18;2021:5542815. doi: 10.1155/2021/5542815 (PMC8238613; doi:10.1155/2021/5542815)
Supplement: Supplementary Materials — Figure S1: constructed from the HSPB1 stable knockout H9c2 cell line and the HSPB1C141S mutant plasmid. Figure S2: the expression of Bmal1 and homooxidized HSPB1 decreased in AMI rat model. Figure S3: homooxidized HSPB1 promoted nuclear transcription of Nrf-2 in H9c2 cells exposed in H2O2. [file 5542815.f1.zip › Supplementary Figures.doc]

**Figure S1**

A	B

C

D

**Figure S2**

A	B

C

**Figure S3**

A	B

C
