# Supplementary material for: Bmal1 Regulates the Redox Rhythm of HSPB1, and Homooxidized HSPB1 Attenuates the Oxidative Stress Injury of Cardiomyocytes
Source: Oxid Med Cell Longev. 2021 Jun 18;2021:5542815. doi: 10.1155/2021/5542815 (PMC8238613; doi:10.1155/2021/5542815)
Supplement: Supplementary Materials — Figure S1: constructed from the HSPB1 stable knockout H9c2 cell line and the HSPB1C141S mutant plasmid. Figure S2: the expression of Bmal1 and homooxidized HSPB1 decreased in AMI rat model. Figure S3: homooxidized HSPB1 promoted nuclear transcription of Nrf-2 in H9c2 cells exposed in H2O2. [file 5542815.f1.zip › Supplementary20210505.doc]

**Supplementary Figure legends**

**Fig. S1.** Constructed of the HSPB1 stable knockout H9c2 cells line and the HSPB1C141S mutant plasmid. (A) LV-HSPB1-RNAi packaged with lentivirus was constructed by GENE company (No: 94459-1; Virus Titer: 1E+9TU/ml). The LV-HSPB1-RNAi was transfected into the H9c2 cells 72 h, and then used the puromycin (1 ug/ml) to screen the cells. Western blot analysis was used to determine the HSPB1 expression. (B) Densitometry was used to determine the fold expression of HSPB1 compared with β-actin. n≥6; ***p < 0.001. (C) RT-PCR used to test the sequence of pEnCMV-HSPB1C141S^mut^ plasmid. (D) H9c2 cells were transfected with pEnCMV-HSPB1C141S^mut^ or vector for 48 h. After, H9c2 cells were treated with 800 mM of H_2_O_2_ for 120 min. NEM-alkylated redox western blotting was performed to detect the redox state of HSPB1. For reducing SDS-PAGE, 5% dithiothreitol was added to the samples (n≥6).

**Fig. S2.** The expression of Bmal1 and homo-oxidized HSPB1 decreased in AMI rat model. Established the sham, AMI and I/R model in SD rats, and then a piece of ventricular muscle come from apex of mice heart were extrcted, (A) and detect Cry, Per2, BMAL1, HSPB1 mRNA levels by real-time quantitative PCR (*P<0.001). (B) and western blot used to test the protein levels of HSPB1, Bmal1 and β-actin. (C) The ventricular apex proteins were extracted using NEM extracting buffer, and the redox status of HSPB1 was detected by non-reducing western blot. For reducing SDS-PAGE, 5% dithiothreitol was added to the samples (n=8).

**Fig. S3.** Homo-oxidized HSPB1 promoted nuclear transcription of Nrf-2 in H9c2 cells exposed in H_2_O_2_. (A) HSPB1 stable knockout H9c2 cell were transfected with 2 ug pEnCMV-HSPB1C141S^mut^ and nomral H9c2 cells were transfected with 2 ug pEnCMV-HSPB1 for 48 h and then treated with different concentrations of H_2_O_2_ (0 or 800 mM) for 60 min. Western blot analysis demonstrated the nuclear and cytoplasmic levels of total Nrf-2, Vinculin, and Laminb1. (B) Relative levels of Nrf-2 compared to Laminb1. (C) Relative levels of Nrf-2 compared to Vinculin. Graphs are representatives of three independent experiments (n≥6; ***p < 0.001).
